# Impact of Equalization-Enhanced Phase Noise on Digital Nonlinearity Compensation in High-Capacity Optical Communication Systems

**DOI:** 10.3390/s20154149

**Published:** 2020-07-26

**Authors:** Jiazheng Ding, Tianhua Xu, Cenqin Jin, Ziyihui Wang, Jian Zhao, Tiegen Liu

**Affiliations:** 1 Key Laboratory of Opto-Electronic Information Technical Science of Ministry of Education, School of Precision Instruments and Opto-Electronics Engineering, Tianjin University, Tianjin 300072, China; whisperil01_@tju.edu.cn (J.D.); zyhwang@tju.edu.cn (Z.W.); tgliu@tju.edu.cn (T.L.); 2 University of Warwick, Coventry CV4 7AL, UK; cenqin.jin@warwick.ac.uk; 3 Department of Electronic and Electrical Engineering, University College London, London WC1E 6BT, UK

**Keywords:** optical fiber communication, electrical dispersion compensation, multi-channel digital backpropagation, equalization-enhanced phase noise, achievable information rates

## Abstract

Equalization-enhanced phase noise (EEPN) can severely degrade the performance of long-haul optical fiber transmission systems. In this paper, the impact of EEPN in Nyquist-spaced dual-polarization quadrature phase shift keying (DP-QPSK), dual-polarization 16-ary quadrature amplitude modulation (DP-16QAM), and DP-64QAM optical transmission systems is investigated considering the use of electrical dispersion compensation (EDC) and multi-channel digital backpropagation (MC-DBP). Our results demonstrate that full-field DBP (FF-DBP) is more susceptible to EEPN compared to single-channel and partial-bandwidth DBP. EEPN-induced distortions become more significant with the increase of the local oscillator (LO) laser linewidth, and this results in degradations in bit-error-rates (BERs), achievable information rates (AIRs), and AIR-distance products in optical communication systems. Transmission systems using higher-order modulation formats can enhance information rates and spectral efficiencies, but will be more seriously degraded by EEPN. It is found that degradations on AIRs, for the investigated FF-DBP schemes, in the DP-QPSK, the DP-16QAM, and the DP-64QAM systems are 0.07 Tbit/s, 0.11 Tbit/s, and 0.57 Tbit/s, respectively, due to the EEPN with an LO laser linewidth of 1 MHz. It is also seen that the selection of a higher-quality LO laser can significantly reduce the bandwidth requirement and the computational complexity in the MC-DBP.

## 1. Introduction

With the explosive growth of the 5G and cloud services, the demand for high-capacity optical fiber communication infrastructure has become unprecedentedly urgent. Researchers have utilized dense wavelength division multiplexing (DWDM) and high-order modulation formats to enhance the spectral efficiency (SE) and the capacity of optical transmission systems. Moreover, Nyquist-spaced superchannel transmission systems, composed of closely-spaced WDM channels spaced at, or close to, the symbol rate, have been applied to further increase the spectral efficiencies [1,2,3,4,5]. However, the chromatic dispersion (CD), the polarization mode dispersion (PMD), the laser phase noise (PN), as well as Kerr fiber nonlinearities (NLs) severely degrade the performance of optical communication systems, leading to constraints on achievable information rates (AIRs), a measure of achievable capacity of communication systems [6,7]. Advancements in powerful digital signal processing (DSP) enhanced the reaches of optical communication systems and achieved the compensation of transmission impairments via the use of electronic dispersion compensation (EDC), digital backpropagation (DBP), and carrier phase estimation (CPE) [8,9,10,11,12,13].

However, in DSP-based coherent systems, the interplay between the laser phase noise and the electronic dispersion compensation can introduce an effect of equalization-enhanced phase noise (EEPN), leading to a significant degradation to the performance of system. A few studies regarding EEPN have been reported for linear optical communication systems, where fiber nonlinearities were neglected. Shieh and Ho have conducted the theoretical derivation and analysis of the generation process of EEPN [14]. Fatadin and Savory have studied the impact of EEPN on the quadrature phase shift keying (QPSK), the 16-ary quadrature amplitude modulation (16-QAM), and the 64-QAM coherent systems using a time-domain dispersion equalizer [15]. Kakkar and Schatz have investigated the effect of the local oscillator (LO) laser linewidth and the symbol rate on the bit-error-rate (BER) of the transmission system [16]. Yoshida and Sugihara simulated the Q-factor of the system under the influence of EEPN by varying the laser linewidth from 100 kHz to 1 MHz [9]. It has been demonstrated that the impact of EEPN scales with the accumulated dispersion, the laser linewidth, the symbol rate, and the modulation format in the communication system.

To compensate for the fiber nonlinearities in optical communication systems, multi-channel digital backpropagation (MC-DBP) has been applied at different bandwidths to mitigate intra-channel and inter-channel fiber nonlinearities, such as self-phase modulation (SPM), cross-phase modulation (XPM), and four-wave mixing (FWM) [13,17,18,19]. The effect of the number of steps per span, the PMD, the backpropagated signal bandwidth, and the LO phase synchronization have been studied in the optimization of MC-DBP [20,21,22]. However, the influence of EEPN on the performance of MC-DBP in optical transmission systems was rarely reported and the impact of EEPN has never been quantified [13]. Therefore, it is of great significance to investigate the effect of EEPN on the MC-DBP-based nonlinear compensation in closely-spaced WDM communication systems.

In this paper, the impact of EEPN on the behaviors of EDC and MC-DBP in 9-channel 32-Gbaud dual-polarization (DP) Nyquist-spaced WDM transmission systems has been investigated, where DP-QPSK, DP-16QAM, and DP-64QAM have been applied. The nonlinearity compensation (NLC) performance using the single-channel DBP, the partial-bandwidth DBP, and the full-field DBP (FF-DBP) were numerically simulated with and without the application of EEPN. The EEPN degradations on AIRs in high-speed optical communication systems have also been studied. It is found that EEPN significantly deteriorates the performance of the communication systems, especially in the case of FF-DBP. Systems using higher-order modulation formats can enhance the AIRs and spectral efficiencies, but will be more seriously deteriorated by EEPN. It is also found that the selection of a better-quality LO laser can greatly reduce the bandwidth requirement and the computational complexity in the MC-DBP.

This paper is organized as follows. In Section 2, the numerical transmission system, the principle of EEPN, the mutual information (MI), and the modulation error ratio (MER) are introduced. In Section 3, simulations and results under different scenarios are analyzed. In Section 4, additional insights and discussions are provided. The conclusions are summarized in Section 5.

## 2. Transmission Setup and Principles

### 2.1. Transmission Setup

**Figure 1:**
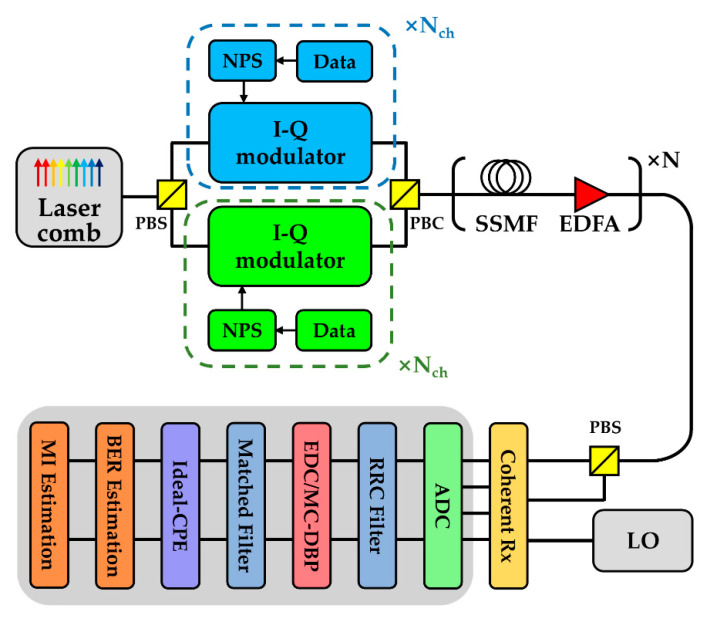
Schematic of the 9-channel 32-Gbaud Nyquist-spaced superchannel transmission system. (NPS: Nyquist pulse shaping, PBS: polarization beam splitter, PBC: polarization beam combiner, LO: local oscillator, CPE: carrier phase estimation, MI: mutual information).

Figure 1 depicts the 9-channel 32-Gbaud Nyquist-spaced superchannel transmission system using the following modulation formats: DP-QPSK, DP-16QAM, DP-64QAM. At the transmitter (Tx), a 9-line 32-GHz spaced laser comb with a central wavelength of 1550 nm is employed as the phase-locked optical carrier for each sub-channel. Each optical carrier is split into two branches using a polarization beam splitter (PBS) and is then fed into two in-phase and quadrature (I-Q) modulators, respectively. The transmitted data sequences in all channels and polarizations are mutually independent based on the use of pseudo-random binary codes (PRBS) with a pattern length of 216. The Nyquist pulse shaping (NPS) is performed using an ideal root-raised cosine (RRC) filter with a roll-off coefficient of 0.1%. After the modulation, two orthogonally polarized signal sequences are combined using a polarization beam combiner (PBC) and are then injected into the standard single-mode fiber (SSMF). Numerical simulation of the SSMF is implemented using the split-step Fourier method based on the nonlinear Schrödinger equation (NLSE) with a step size of 0.1 km. Simulation parameters of SSMF include an attenuation coefficient of 0.2 dB/km, CD coefficient of 17 ps/nm/km, and a nonlinear coefficient of 1.2 /w/km, which represents a typical set of SSMF according to the Corning SMF-28 datasheet. An erbium-doped fiber amplifier (EDFA) is used to compensate for the loss of fiber in each span during the transmission. The noise figure of EDFA is 5.0 dB, which is a typical value for standard EDFA [1]. The fiber length of each span is 80 km. At the receiver (Rx), the received signal is mixed with the LO laser to achieve an ideal coherent detection of I-Q components in each polarization state. The signals are sampled using analog-to-digital converters (ADCs) and are then processed by DSP modules which include a dispersion compensation module using EDC, nonlinearity compensation module using MC-DBP, matched filter, CPE, and symbol demodulation. In our simulation, the frequency domain equalizer (FDE) is used for CD compensation [23], and the MC-DBP is used for NLC [24]. The MC-DBP algorithm is implemented based on the reverse split-step Fourier solution of the NLSE and the compensation bandwidth is set as 32 GHz (for 1-channel DBP), 160 GHz (for 5-channel DBP), and 288 GHz (for 9-channel DBP, FF-DBP), respectively. Before the MC-DBP module, an ideal RRC filter with a roll-off of 0.1% is applied to select the compensation bandwidth and to remove the unwanted amplifier spontaneous emission noise (ASE). An ideal CPE is used for the compensation of phase noise, achieved using the conjugate multiplication between the received signal and the extracted intrinsic laser phase noise [13,25]. This is to isolate the impact from EEPN, where no amplitude noise mitigation effect is employed in the CPE module [15,26]. BER and MI estimation are carried out to evaluate the transmission performance. The PMD and the frequency offset are neglected in all simulations.

### 2.2. Principle of Equalization Enhanced Phase Noise

As illustrated in Figure 1, the EDC is used to compensate for dispersion, and the ideal CPE is applied for the CPE in the coherent optical transmission system. The dispersion compensation module (either EDC or MC-DBP) is applied prior to the CPE module [13]. It can be clearly seen that the phase noise from the transmitter passes through the transmission fiber and the dispersion compensation module, so the net dispersion experienced by the phase noise from the transmitter is close to zero. However, the phase noise from the LO laser only goes through the dispersion compensation module. Due to the non-commutability of the phase noise and fiber dispersion, the phase noise of the LO laser will interact with the DSP-based dispersion compensation module. Consequently, the electronic equalizers (EDC or MC-DBP) will enhance the distortions from the LO laser phase noise, and the resulting EEPN will seriously reduce the performance of the large-capacity transmission system.

It has been reported in [11,14,27] that the variance of EEPN has a linear relationship with the accumulated chromatic dispersion, the linewidth of LO laser, and the symbol rate in the system. The noise variance generated by EEPN can be expressed as [14]

(1) $$\sigma_{EEPN}^{2}=\frac{\pi \lambda^{2}D\cdot L\cdot \Delta f_{LO}}{2cT_{S}}$$

where *λ* is the center wavelength of the carrier laser, *D* is the dispersion coefficient of SSMF, *L* is the total length of fiber link, $\Delta f_{LO}$ is the 3-dB linewidth of the LO laser, *c* is the speed of light in vacuum, and $T_{S}$ is the symbol period of the transmission system.

Therefore, the total noise variance of coherent transmission system considering the impairment of EEPN is expressed as follows:

(2) $$\sigma_{T\mathrm{ot}al}^{2}=\sigma_{Tx}^{2}+\sigma_{LO}^{2}+\sigma_{EEPN}^{2}+2\rho \sigma_{LO}\sigma_{EEPN}$$

(3) $$\sigma_{Tx}^{2}=2\pi \Delta f_{Tx}T_{S}$$

(4) $$\sigma_{LO}^{2}=2\pi \Delta f_{LO}T_{S}$$

where $\sigma_{Tx}^{2}$ and $\sigma_{LO}^{2}$ are the variances of the phase noise from the transmitter and receiver, respectively, ρ is the correlation coefficient of the LO phase noise and the EEPN. It has been proved that ρ≈0 when the length of the optical fiber link exceeds 80 km [11]. Therefore, in a high-speed and long-distance superchannel optical communication system, the total noise variance can be approximately expressed as

(5) $$\sigma_{T\mathrm{ot}al}^{2}=\sigma_{Tx}^{2}+\sigma_{LO}^{2}+\sigma_{EEPN}^{2}$$

It is noted that when the ideal CPE is applied, the intrinsic phase noise $\sigma_{Tx}^{2}$ and $\sigma_{LO}^{2}$ will be fully removed.

### 2.3. Mutual Information and Achievable Information Rate Estimation

Due to the property of the binary symmetric channel, the hard-decision forward error correction (HD-FEC) based code rate of the QPSK and m-QAM signal transmission over an additive white Gaussian noise (AWGN) channel can be expressed as [28,29]

(6) $$R_{C}=1+BER\cdot \log_{2}(BER)+(1-BER)\cdot \log_{2}(1-BER)$$

In communication systems, mutual information is an indicator of the achievable channel capacity under specific modulation schemes. In dual-polarization coherent optical transmission systems, the MI can be expressed as

(7) $$MI=2\cdot \log_{2}(m)\cdot R_{C}$$

where *m* is the order of the modulation format. The AIR is also an important indicator, which refers to the net data rate that can be achieved using FEC decoders [30]. In a dual-polarization Nyquist-spaced optical transmission system, AIRs are expressed by MI as

(8) $$AIR=N_{\mathrm{ch}}\cdot R_{S}\cdot MI$$

Where $N_{\mathrm{ch}}$ is the number of WDM channels in the system, and $R_{S}$ is the symbol rate of the transmitted signal.

### 2.4. Modulation Error Ratio

The MER is also an important indicator to evaluate the quality of a communication system, and it can well reflect the ability of digital receivers in restoring transmitted data [31]. The MER is measured using the ratio between the average signal power and the average error power [32], and is defined in dB as follows

(9) $$MER(dB)=10\log_{10}(\frac{P_{signal}}{P_{error}})$$

Where $P_{error}$ is the root mean square (RMS) power of the error vector and $P_{signal}$ is the RMS power of the ideal transmitted signal. It can be found that the MER provides a similar evaluation on the quality of the received signal, compared to the concept of the signal-to-noise-ratio (SNR) [4].

## 3. Simulation Results

**Figure 2:**
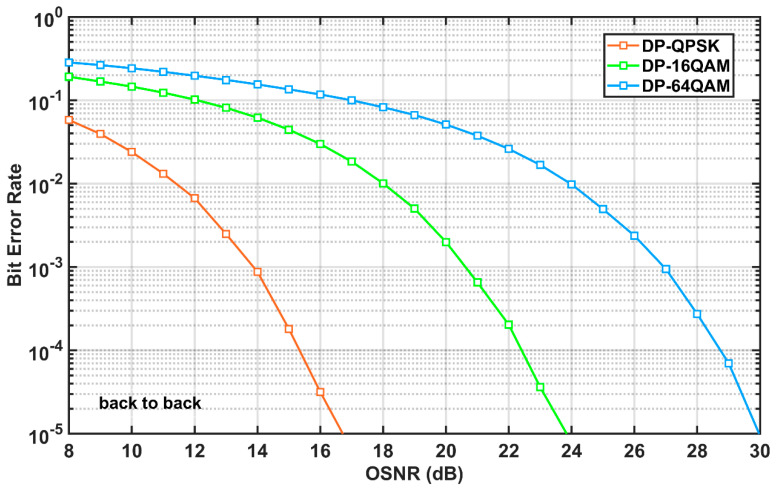
Back-to-back (BtB) performance of 9-channel 32-Gbaud Nyquist-spaced dual-polarization quadrature phase shift keying (DP-QPSK), dual-polarization 16-ary quadrature amplitude modulation (DP-16QAM), and dual-polarization 64-ary quadrature amplitude modulation (DP-64QAM) transmission systems.

In this section, numerical simulations based on the above analyses will be conducted. Firstly, the back-to-back (BtB) performance is investigated to evaluate the benchmark quality of considered transmission systems. Figure 2 shows the BtB performance of the measured BER versus the optical signal-to-noise-ratio (OSNR) for the 9-channel 32-Gbaud Nyquist-spaced DP-QPSK, DP-16QAM, and DP-64QAM systems. It is found that, to achieve a BER target of $10^{-3}$, the required OSNRs in the DP-QPSK, the DP-16QAM, and the DP-64QAM transmission systems are 14.0 dB, 20.6 dB, and 27.0 dB, respectively.

With and without taking EEPN into account, the BERs and AIRs of the 9-channel Nyquist-spaced transmission systems using EDC and MC-DBP are studied. Modulation formats of DP-QPSK, DP-16QAM, and DP-64QAM were applied. The linewidths of the transmitter laser and the LO laser were both set as 0 Hz to remove the EEPN. To include the EEPN, both the transmitter and LO laser linewidths were set to be equal, and two sets of values were considered: 100 kHz (for common ECL lasers) and 1 MHz (for common DFB lasers), respectively. Single-channel, partial-bandwidth, and full-field DBP were applied to perform nonlinearity compensation in the DSP-based receiver. The case of CD compensation only was also analyzed as reference results. In all following simulations, the MC-DBP was operated with 800 steps per span and a nonlinear coefficient of 1.2 /W/km to ensure its optimal performance.

**Figure 3:**
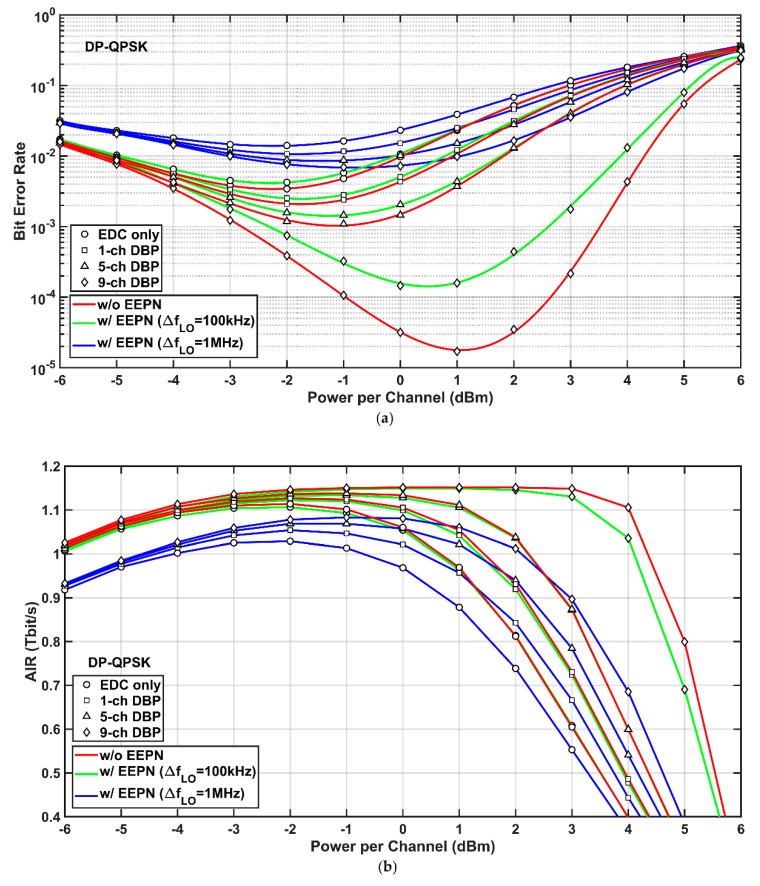
Simulation results of the DP-QPSK system with (w/) and without (w/o) equalization-enhanced phase noise (EEPN) at different multi-channel digital backpropagation (MC-DBP) bandwidths. (**a**) Bit-error-rate (BER) versus optical launch power, (**b**) achievable information rate (AIR) versus optical launch power.

Simulation results of the 9-channel 32-Gbaud Nyquist-spaced DP-QPSK transmission system with and without the application of EEPN are shown in Figure 3, where the total distance of fiber link is 8000 km. The results are obtained under the use of EDC or MC-DBP with different back-propagated bandwidths. Figure 3a shows the BER versus optical launch power in DP-QPSK transmission system, the system BER using MC-DBP is obviously reduced compared with the EDC-only scheme under different LO linewidths. The compensation effect of FF-DBP is the most obvious because the intra-channel and inter-channel nonlinear interactions in the transmission channels were fully mitigated. When there is no EEPN, the optimum launch powers of −2.0 dBm, −2.0 dBm, −1.0 dBm, and 1.0 dBm with the best BERs of 3.43 × 10^−3^, 2.10 × 10^−3^, 1.09 × 10^−3^, and 1.70 × 10^−3^ are achieved for the scenarios of EDC, single-channel, 5-channel, and full-field DBP, respectively. However, when EEPN is applied with an LO laser linewidth of 100 kHz, the optimum launch powers for EDC, signal-channel, and 5-channel DBP are still −2.0 dBm, −2.0 dBm, and −1.0 dBm, respectively. However, the optimum launch powers for full-field DBP is changed to 0.0 dBm. Corresponding BERs rise to 4.25 × 10^−3^, 2.54 × 10^−3^, 1.45 × 10^−3^, and 1.46 × 10^−3^ compared to the scenario of no EEPN. When the LO laser linewidth rises to 1 MHz, the distortions from EEPN will become greater according to Equation (1). Simulation results in Figure 3a show that the optimum launch powers for EDC, signal-channel, and 5-channel DBP remain constant, respectively, but for FF-DBP, it drops to −1.0 dBm. The system BERs increase further to 1.41 × 10^−2^, 1.07 × 10^−2^, 8.75 × 10^−3^, and 6.95 × 10^−3^ for EDC, signal-channel, 5-channel, and full-field DBP, respectively. It can be clearly seen that the performance of FF-DBP is the most susceptible to EEPN compared to EDC, single-channel, and 5-channel DBP. Due to the EEPN (with an LO laser linewidth of 1 MHz), the best BER increases by two and a half orders of magnitude (from 1.70 × 10^−5^ to 6.95 × 10^−3^) in the case of FF-DBP, while it only increases by less than an order of magnitude (from 3.43 × 10^−3^ to 1.41 × 10^−2^) in the case of EDC.

The relationship between the AIRs and optical launch powers in the 9-channel 32-Gbaud Nyquist-spaced DP-QPSK optical communication system was investigated, as described in Figure 3b. When there is no EEPN, the highest AIRs for the system are 1.11 Tbit/s, 1.13 Tbit/s, 1.14 Tbit/s, and 1.15 Tbit/s for the cases of EDC, single-channel, 5-channel, and full-field DBP, respectively. When the EEPN is applied with an LO linewidth of 100 kHz, it can be seen from Figure 3b that the AIRs of the transmission system are not significantly reduced in all compensation schemes for such an amount of EEPN. However, the degradation on AIRs becomes significant when the LO laser linewidth increases to 1 MHz. The best AIRs now become 1.03 Tbit/s, 1.05 Tbit/s, 1.07 Tbit/s, and 1.08 Tbit/s for the cases of EDC, single-channel, 5-channel, and full-field DBP, respectively. It is noted that, for all launch powers smaller than 0.5 dBm, the AIRs in the cases of single-channel and 5-channel DBP with the LO laser linewidth of 100 kHz are higher than the AIRs in the case of FF-DBP with the LO laser linewidth of 1 MHz. This indicates that a better-quality LO laser, which has a narrower linewidth and leads to a smaller EEPN, can significantly save the bandwidth and the computational effort in the operation of MC-DBP.

**Figure 4:**
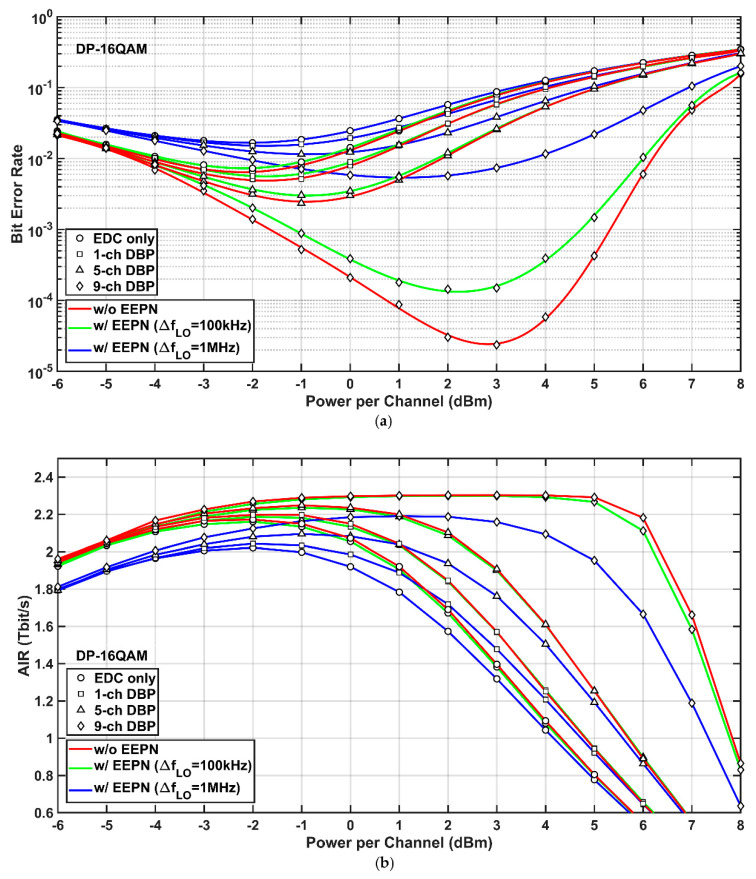
Simulation results of the DP-16QAM system with (w/) and without (w/o) EEPN at different MC-DBP bandwidths. (**a**) BER versus optical launch power, (**b**) AIR versus optical launch power.

Figure 4 shows the simulation results of the 9-channel 32-Gbaud Nyquist-spaced DP-16QAM system at different scenarios of MC-DBP with and without the application of EEPN. The transmission distance is 2000 km. It can be seen from Figure 4a that the optimum launch powers for the use of EDC, single-channel, partial-channel, and full-field DBP are −2.0 dBm, −2.0 dBm, −1.0 dBm, 3.0 dBm, respectively, when there is no EEPN. Corresponding optimal BERs are 6.56 × 10^−3^, 5.11 × 10^−3^, 2.35 × 10^−3^, and 2.36 × 10^−3^, respectively. When the EEPN is applied with an LO laser linewidth of 1 MHz, the optimum launch powers keep the same for the cases of EDC, single-channel, and 5-channel DBP, but decrease to 1.0 dBm for the case of FF-DBP. Meanwhile, the optimal BERs rise to 1.67 × 10^−2^, 1.51 × 10^−2^, 1.51 × 10^−2^, and 5.51 × 10^−3^, respectively. Due to the EEPN with an LO laser linewidth of 1 MHz, the best BER for the FF-DBP increases by two and a half orders of magnitude, similar as the DP-QPSK system. Apparently, the DP-16QAM system is more sensitive to the EEPN than the DP-QPSK system. Because the transmission distance in the DP-16QAM system is only a quarter of that in the DP-QPSK system, this will lead to the reduction of EEPN variance (to a quarter) according to Equation (1).

The AIRs versus optical launch powers in the 9-channel 32-Gbaud Nyquist-spaced DP-16QAM transmission system are shown in Figure 4b. It is found from Figure 4b that, similar to the DP-QPSK system, the EEPN with an LO laser linewidth of 100 kHz poses marginal degradation on AIRs in all EDC and MC-DBP schemes compared to the scenario without any EEPN. However, the EEPN with an LO laser linewidth of 1 MHz will cause a significant degradation to the AIRs. For the FF-DBP, the best AIR drops from 2.30 Tbit/s to 2.19 Tbit/s and the optimum launch power decreases from 3.0 dBm to 1.0 dBm compared to the scenario without any EEPN. It is noted again that, for launch powers smaller than 1.0 dBm, the AIRs in the case of single-channel DBP with the LO laser linewidth of 100 kHz are higher than the AIRs in the case of 5-channel DBP with the LO laser linewidth of 1 MHz, and the AIRs in the case of 5-channel DBP with the LO laser linewidth of 100 kHz are higher than the AIRs in the case of FF-DBP with the LO laser linewidth of 1 MHz. This again demonstrates that the choice of a better-quality LO laser can significantly reduce the bandwidth requirement and the computational complexity in the MC-DBP.

**Figure 5:**
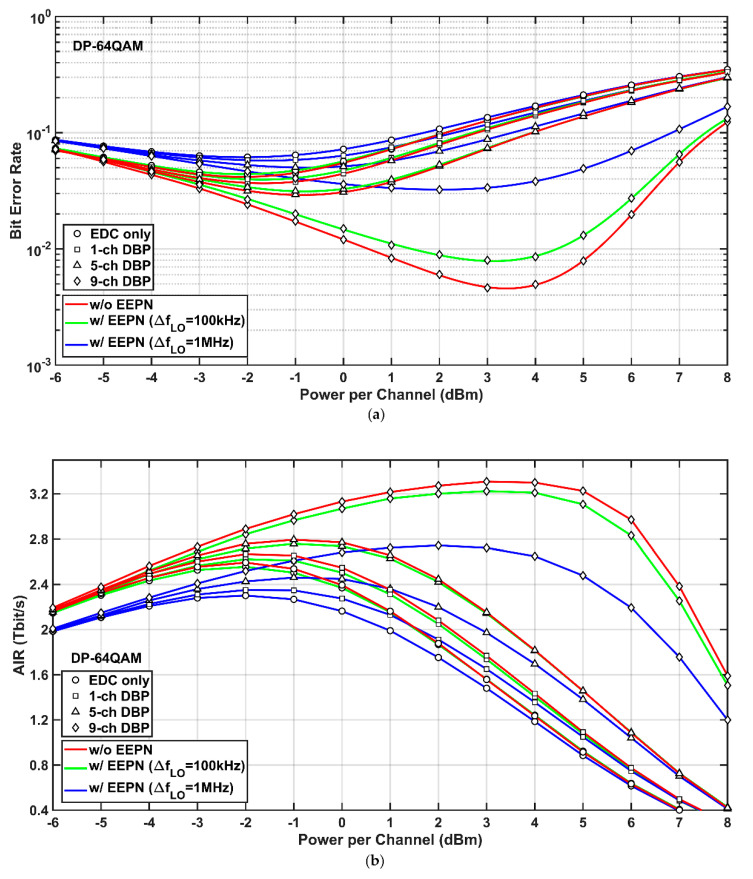
Simulation results of the DP-64QAM system with (w/) and without (w/o) EEPN at different MC-DBP bandwidths. (**a**) BER versus optical launch power, (**b**) AIR versus optical launch power.

Simulation results of the 9-channel 32-Gbaud Nyquist-spaced DP-64QAM transmission system using EDC and MC-DBP with and without the application of EEPN are illustrated in Figure 5, where the transmission distance is 1200 km. Figure 5a describes that BERs versus optical launch powers in the DP-64QAM superchannel transmission, which shows a similar trend as the BERs the DP-QPSK and the DP-16QAM systems. Due to the EEPN with an LO laser linewidth of 1 MHz, the optimum launch power for the FF-DBP drops from 3.5 dBm to 2.0 dBm, and the best BER increases from 4.63 × 10^−3^ to 3.23 × 10^−2^ compared to the scenario without any EEPN. This is similar as the BER degradation in the DP-QPSK and the DP-16AM system. This also indicates that the DP-64QAM system is more vulnerable to the EEPN than the DP-QPSK and the DP-16QAM systems, since the transmission distance in the DP-64QAM system is only 3/20 of the transmission distance in the DP-QPSK system and 3/5 of the transmission distance in the DP-16QAM system. This will lead to a significant reduction of EEPN variance compared to that in the DP-QPSK and the DP-16QAM systems according to Equation (1). Therefore, the EEPN will pose greater distortions on the systems with higher-order modulation formats, resulting in a stricter requirement on laser linewidths, although they can improve the transmission spectral efficiencies.

Simulation results of AIRs versus optical launch powers for the 9-channel 32-Gbaud Nyquist-spaced DP-64QAM system at different MC-DBP bandwidths, with and without the application of EEPN, are shown in Figure 5b. Again, similar to the DP-QPSK and the DP-16QAM systems, the EEPN with an LO laser linewidth of 100 kHz puts marginal degradation on AIRs in all schemes of EDC and MC-DBP, compared to the scenario without any EEPN. The EEPN with an LO laser linewidth of 1 MHz will pose a significant degradation to the AIRs of the DP-64QAM system. For the FF-DBP, the best AIR decreases from 3.31 Tbit/s to 2.74 Tbit/s. Meanwhile, a similar effect can be found that, for launch powers smaller than 0.5 dBm, the AIRs in the use of single-channel DBP with the LO laser linewidth of 100 kHz are higher than the AIRs in the use of 5-channel DBP with the LO laser linewidth of 1 MHz, and the AIRs in the use of 5-channel DBP with the LO laser linewidth of 100 kHz are higher than the AIRs in the use of FF-DBP with the LO laser linewidth of 1 MHz. This again implies that the use of a better-quality LO laser can significantly reduce the bandwidth requirement and the computational complexity in the MC-DBP. It can also be found in the AIR results that the DP-64QAM system is more susceptible to the EEPN compared to the DP-QPSK and the DP-16QAM systems, since the AIR degradation (~0.57 Tbit/s) for the case of FF-DBP in the DP-64QAM system due to the EEPN with the LO laser linewidth of 1 MHz is more significant than those (~0.11 Tbit/s and ~0.07 Tbit/s, respectively) in the DP-QPSK and the DP-16QAM systems, although the DP-64QAM system has the shortest transmission distance. In addition, the increase in EEPN due to the increase of the LO laser linewidth shows a smaller effect on the AIR degradations in single-channel DBP and partial-bandwidth DBP than in FF-DBP.

## 4. Discussions

**Figure 6:**
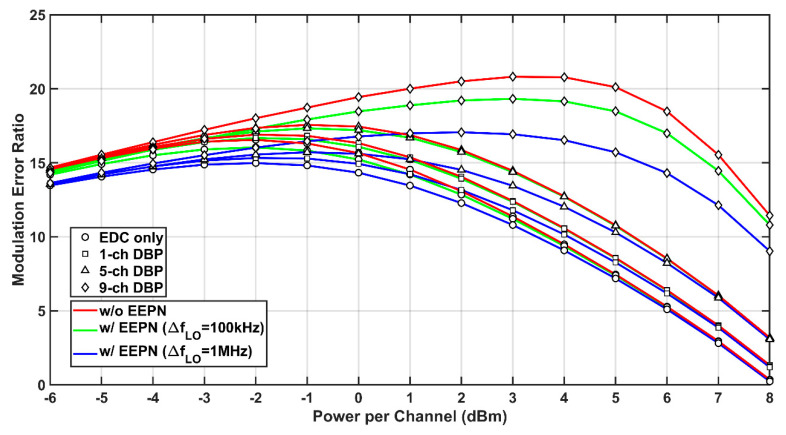
Modulation error ratio (MER) versus optical launch power in the DP-64QAM system with (w/) and without (w/o) EEPN at different MC-DBP bandwidths.

The MER reflects the ability of digital receivers in restoring transmitted data. Considering the EEPN, the relationship between the MER and the optical launch power in the DP-64QAM communication system using EDC and MC-DBP is investigated in Figure 6, where the transmission distance is 1200 km. It is found that, without EEPN, the maximum MER is 20.81 dB in the case of FF-DBP when the optical launch power is 3 dBm. However, the MER will be reduced to 17.06 dB when the LO laser linewidth is increased to 1 MHz, and the optimal launch power will be reduced to 2 dBm accordingly. It can be found that the EEPN degradations on MERs are also more serious in the case of FF-DBP than those in the scenarios of the EDC, the single-channel, and the partial-bandwidth DBP. This shows a consistent behavior compared to previous analyses.

**Figure 7:**
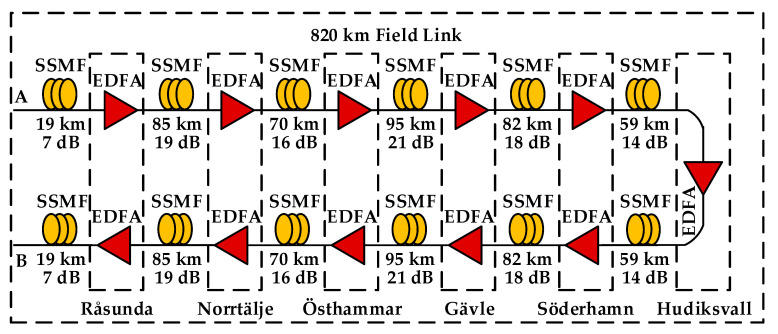
Block diagram of 820 km installed fiber field link in the eastern coast of Sweden.

**Figure 8:**
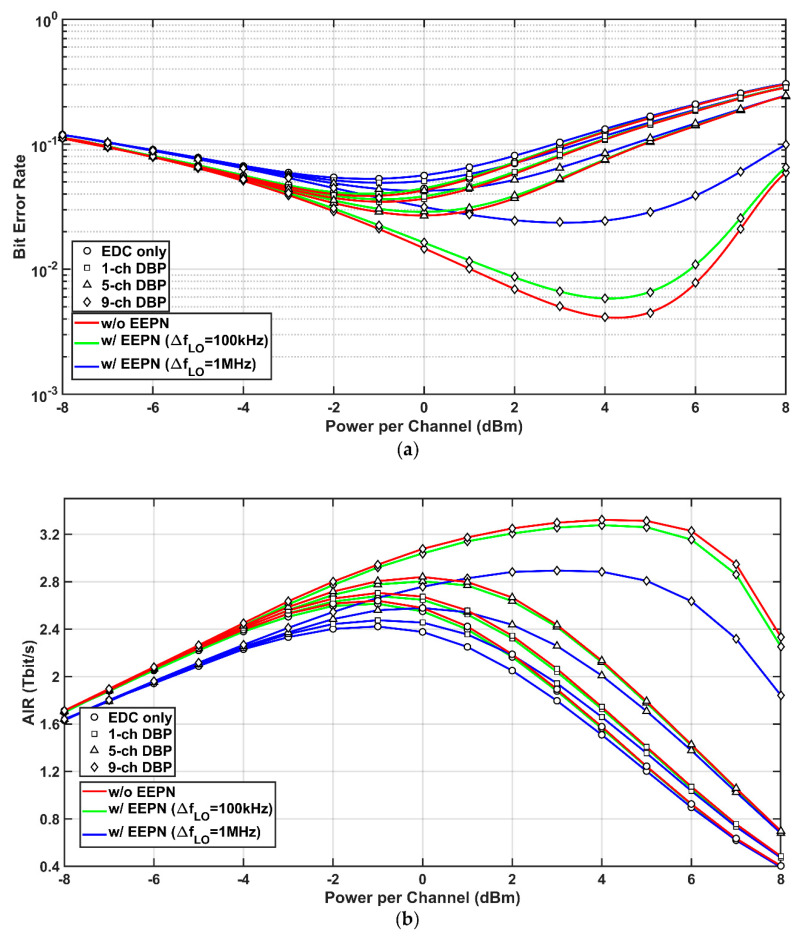
Simulation results of the DP-64QAM system with (w/) and without (w/o) EEPN at different MC-DBP bandwidths in the 820 km field link. (**a**) BER versus optical launch power, (**b**) AIR versus optical launch power.

To provide a more practical study on the impact of EEPN in optical fiber communication systems, numerical simulations have been carried out based on an 820 km optical fiber field link installed in the eastern coastal area of Sweden. Figure 7 illustrates the block diagram of the 820 km field link, which interconnects seven nodes at Kista, Råsunda, Norrtälje, Östhammar, Gävle, Söderhamn, and Hudiksvall [33]. The transmitter, the receiver, and the DSP setups are the same as those employed in Figure 1. The impact of EEPN on the performance of EDC and MC-DBP in the 820 km transmission system is investigated, as shown in Figure 8, where the modulation format of DP-64QAM is applied. Similar to previous analyses, it can be found that the EEPN also poses significant degradations on the performance of the MC-DBP for such a practical transmission link. For the FF-DBP and the EEPN with an LO laser linewidth of 1 MHz, the optimum launch power drops from 4.0 dBm to 3.0 dBm, and the best BER increases from 4.15 × 10^−3^ to 2.39 × 10^−2^, and the best AIR decreases from 3.32 Tbit/s to 2.89 Tbit/s, compared to the scenario without any EEPN.

From the above analyses, the intrinsic phase noise from the transmitter and LO lasers in the transmission system was completely compensated by the ideal CPE in the DSP, so the degradations in the system AIRs come from EEPN and the fiber NLs. From the aforementioned results, FF-DBP is the most susceptible to EEPN compared to the single-channel and the partial-bandwidth DBP. This is because the phase change in the DBP (actually the dispersion compensation) filter increases quadratically with the increase of the signal frequency [13,14]. Consequently, EEPN will pose a more serious degradation in the case of FF-DBP. The distortions from EEPN also become more significant for a larger LO linewidth, resulting in a larger increase of BER and a larger reduction in the optimum launch power of FF-DBP. Therefore, in high-capacity optical communication systems, EEPN put strict requirements on the linewidths of the LO lasers, and the restrictions are stronger for the systems using higher-order modulation formats.

**Figure 9:**
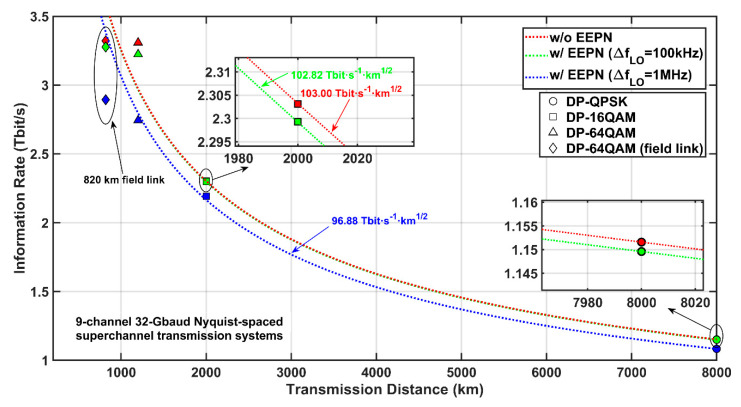
Maximum AIRs versus transmission distance for the DP-QPSK, the DP-16QAM, the DP-64QAM, and the 820 km field-link Nyquist-spaced transmission systems with and without EEPN. The three hyperbolas are obtained by $AIR\cdot \sqrt{L}=C$ based on the 9-channel 32-Gbaud Nyquist-spaced DP-QPSK system under the transmission distance of 8000 km and three LO laser linewidths (0 Hz, 100 kHz, 1 MHz).

The use of higher-order modulation formats can improve the spectral efficiency in optical communication systems. However, it can be seen from the above simulation results that the systems using higher-order modulation formats are more seriously distorted by EEPN. Figure 9 illustrates the maximum AIRs versus transmission distances for the DP-QPSK, the DP-16QAM, the DP-64QAM, and the field-link Nyquist-spaced transmission systems with and without the application of EEPN. Here, the product between AIR and transmission distance $AIR\cdot \sqrt{L}$ is employed as an indicator to evaluate the transmission quality of optical communication systems, the three hyperbolas in Figure 9 are provided using the formula of $AIR\cdot \sqrt{L}=C$ based on the 9-channel 32-Gbaud Nyquist-spaced DP-QPSK system under the transmission distance of 8000 km and three LO laser linewidths (0 Hz, 100 kHz, 1 MHz). 

The AIR-distance product can reach 103.00 Tbit s^−1^km^1/2^ and 102.82 Tbit s^−1^km^1/2^ for the DP-QPSK system when the LO linewidths are 0 Hz and 100 kHz, respectively. However, it can only achieve 96.88 Tbit s^−1^km^1/2^ when the LO linewidth is 1 MHz. It is very interesting that the AIR-distance products of the 9-channel 32-Gbaud Nyquist-spaced DP-16QAM system are basically equal to the AIR-distance products in the DP-QPSK system for all different values of EEPN. For the 9-channel 32-Gbaud Nyquist-spaced DP-64QAM system, the AIR-distance products are 114.63 Tbit s^−1^km^1/2^ and 111.68 Tbit s^−1^km^1/2^ for the LO linewidths of 0 Hz and 100 kHz, respectively. This shows a better performance (based on the indicator of the AIR-distance product) compared to the DP-QPSK and the DP-16QAM systems. However, the product drops to 95.05 Tbit s^−1^km^1/2^ when the LO linewidth rises to 1 MHz, which is similar to the indicators in the DP-QPSK and the DP-16QAM systems. For the 820 km field-link transmission, the system shows a significant performance degradation compared to all above three systems in terms of the AIR-distance product. It can only reach 82.88 Tbit s^−1^km^1/2^ in the case of FF-BDP when the LO laser linewidth is 1 MHz.

In this research, the impact of EEPN is investigated in optical communication systems based on the use of MC-DBP, which is one of the most promising approaches for nonlinearity compensation in next-generation optical fiber networks, due to its flexible implementation and optimization in the hardware. Meanwhile, the optical phase conjugation (OPC) is another promising technique to compensate for fiber nonlinearities in WDM optical transmission systems [34,35]. In such systems, the phase fluctuation from the high-power pump laser, which is used for realizing the FWM-based OPC, will be transferred into the phase of output signals [13]. Therefore, the EEPN in OPC-based systems arises from the interactions between the phase noise from the high-power pump laser and the fiber dispersion experienced by the phase-conjugated optical signals.

## 5. Conclusions

In this paper, the impact of EEPN on both BER performance and AIRs of Nyquist-spaced superchannel transmission system have been investigated based on the use of EDC and MC-DBP. Different modulation formats including DP-QPSK, DP-16QAM, DP-64QAM have been applied. Distortions from EEPN are more significant in the transmission system when the LO linewidth increases. This results in a reduction in both the AIRs and the AIR-distance products. It is shown that the FF-DBP is more susceptible to EEPN compared to the scenarios of EDC, single-channel, and partial-bandwidth DBP. For the investigated FF-DBP schemes, AIRs are reduced by ~0.07 Tbit/s, ~0.11 Tbit/s, and ~0.57 Tbit/s in the DP-QPSK, the DP-16QAM, and the DP-64QAM systems, respectively, due to the EEPN with an LO laser linewidth of 1 MHz. Systems using higher-order modulation formats will improve the AIRs and spectral efficiencies, but will be more susceptible to EEPN. It is also found that the choice of a better-quality LO laser can significantly reduce the bandwidth requirement and the computational complexity in the MC-DBP. Our research provides a novel insight in the design of long-haul high-capacity optical communication systems using multi-channel nonlinearity compensation techniques.
